# Itaconic Acid Based Surfactants: I. Synthesis and Characterization of Sodium *n*-Octyl Sulfoitaconate Diester Anionic Surfactant

**DOI:** 10.1007/s11743-015-1769-4

**Published:** 2016-02-01

**Authors:** Jun Xu, Fengzhi Cao, Tong Li, Shuai Zhang, Chuanhui Gao, Yumin Wu

**Affiliations:** Chemical Engineering College, Qingdao University of Science and Technology, Qingdao, 266042 People’s Republic of China

**Keywords:** Sodium *n*-octyl sulfoitaconate diester, Sulfonation, Esterification, Anionic surfactant

## Abstract

A novel itaconate-based surfactant, namely sodium *n*-octyl sulfoitaconate diester (SOSID), has been synthesized from itaconic acid (IA) and *n*-octanol by sulfonation and esterification reaction processes. The effects of reaction temperature, reaction time, molar ratios of *n*-octanol to IA and the catalyst dosage on the esterification were investigated. The chemical structure of the surfactants SOSID was characterized by means of LC–MS and confirmed by FT-IR and ^1^H NMR spectroscopy. The surface tension *γ* and the critical micelle concentration (CMC) were determined as 25.02 mN/m and 4.0 × 10^−4^ mol/L by using surface tensiometer at 20 °C. Further investigations showed that SOSID possess excellent wetting, emulsifying and lime soap dispersing properties.

## Introduction

During the last two decades there has been a growing interest in developing novel anionic surfactants with outstanding physicochemical properties. For example, anionic surfactant Aerosol-OT (AOT, sodium bis(2-ethylhexyl) sulfosuccinate) has been extensively investigated due to its exceptional efficiency in forming reverse micelles and in entrapping large amounts of water [1–3]. Compared with the conventional surfactants with one head group and one long-chain alkyl group, AOT-like surfactants with one head group and two long-chain alkyl groups generally present a lower critical micelle concentration (CMC), lower surface tension measured at the CMC, lower Krafft point and preferable solubility.

At present, the starting materials used for preparation of commercial surfactants come predominantly from fossil resources. Because of the limited supply and environmental concerns caused by utilization of fossil resources, it has gained growing interest of chemists to synthesize surfactants from renewable resources [4, 5]. Itaconic acid (IA) is an unsaturated dicarboxylic acid with conjugated double bonds, which can be industrially produced through the fermentation from starch. Typically, IA was listed as one of the “top value-added chemicals from bio-mass” by the US Department of Energy [6]. Due to their unique structures and characteristics, IA and its derivatives can be used as the starting materials for synthesis of polymers [7], such as fiber, plastics, rubber, paints, ion-exchange resins and lubricant [8]. Furthermore, IA has been utilized substantially in surfactant-free emulsion polymerization to generate stable latexes [9, 10]. As a polymerizable dicarboxylic acid, it shows great potential in synthesis of polymerizable surfactants. Up to date, however, there are few reports related to the synthesis of itaconate-based surfactants. In 2005, Prasath et al. [11] prepared nonionic polymerizable surfactants using itaconic anhydride and appropriate long-chain alcohols as starting materials. Two of itaconate-based surfactants, namely monododecyl itaconate (MDDI) and monocetyl itaconate (MCI), have been prepared and used in the batch emulsion polymerization of styrene. We recently reported the preliminary study of sulfoitaconate ester surfactants, namely sodium nonylphenol ethoxylate (10) sulfoitaconate monoester and diester, prepared by esterification of nonylphenol ethoxylate (10) ester (NP-10) with itaconic acid and then sulfonation with sodium sulfite [12]. Because the sulfonation of itaconate diester is a heterogeneous reaction, a phase transfer catalyst is needed to reduce the reaction time, but the purity of surfactant decreases with the addition of catalyst. In the present paper, a process of sulfonation followed by esterification is described which could avoid the presence of heterogeneous reactions. A novel itaconic acid-based anionic surfactant is prepared by the modified process using IA, sodium bisulfite and *n*-octanol as the starting materials. The synthesis and characterization of the itaconate-based surfactant is discussed. The itaconate-based surfactant is obtained in good yield and high purity, and shows high surface activity.

## Experimental

### Materials

Itaconic acid (IA, C.R. Grade, Langyatai Group Co., Ltd., China), *n*-octanol (A.R. Grade, Sinopharm chemical Reagent Co., Ltd. China), Sodium bisulfite (A.R. Grade, Tianjin Bodi Chemical Reagent Factory, China) and *p*-toluenesulfonic acid (A.R. Grade, Tianjin Bodi Chemical Reagent Factory, China) were used as received. Deionized water was used in the synthesis. Mass spectrometry was performed in a LC–MS/MS Waters ACQUIY (liquid chromatography linked to tandem mass spectrometry) instrument fitted with an electrospray interface (ESI), and controlled by MassLynx software. The mass spectrometer was operated in the negative ion mode with LTQ-Orbitrap XL detection; samples (5 mg/L) were injected directly into the mass spectrometer at a flow rate of 5 μL/min. The flow phases were acetonitrile and 5 mmol/L aqueous ammonium acetate solution. Nitrogen flow for nebulizer was at 3.3 L/min (10 arb). Capillary temperature was 350 °C [13].

### Surfactant Synthesis

#### Synthesis of Sodium of Sulfoitaconate (SSI)

Sodium bisulfite, NaHSO_3_ (20.80 g, 0.20 mol), was dissolved in water (100 mL). Itaconic acid (28.60 g, 0.22 mol) was added carefully to the NaHSO_3_ solution. The mixture was put in a 250 mL four-neck flask equipped with a refluxing condenser under constant stirring at 90 °C for 5 h under a nitrogen atmosphere, and it was finally cooled to room temperature. Then hydrochloric acid (0.1 mol) was added to the mixture and it was stirred for 1 h. The precipitate was separated from the solution and dried at 80 °C for 12 h. The obtained dry powder was washed carefully with ethanol of 60 mL for five times. After a separation, the precipitate was dried in vacuum for 24 h. Finally, SSI (37.99 g, 83.27 %) in form of white powder was obtained and used in the following esterification step without further purification.

#### Synthesis of Sodium *n*-Octyl Sulfoitaconate Diester (SOSID)

**Scheme 1:**
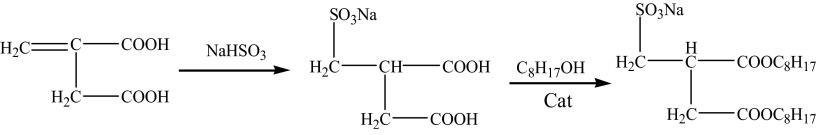
Synthesis of sodium *n*-octyl sulfoitaconate diester (SOSID)

A mixture of Sodium Sulfoitaconate (SSI, 10.60 g, 0.05 mol), *n*-Octanol (13.33 g, 0.10 mol) and *p*-toluene sulphonic acid (0.42 g, 2.44 mmol) in a 100 mL four-neck round-bottom flask with a refluxing condensed was stirred at 140 °C for 4 h under nitrogen, and then cooled to room temperature. The solid residue was treated with petroleum ether (5 × 30 mL) and acetone (5 × 30 mL) successively. After separation of the solvents, the white powder was dried in vacuum for 12 h and then SOSID (18.69 g, 89.86 %) was obtained (Scheme 1).

The sulfonation yield of the itaconic acid has been confirmed by measuring the iodine content in the reaction system according to the Chinese standard GB/T 13892-2012. The yield calculation is represented by the following Eq. (1):

1 $${\text{Yield}}_{1} = \frac{Y - X}{Y} \times 100\,\%$$

where *X* and *Y* denote the final iodine content and the initial iodine content in the reaction system, respectively.

The esterification yield of the derivatives of sulfonated itaconic acid was determined by means of detecting the acid content in the reaction system according to the Chinese standard GB/T 5530-2005/ISO 660: 1996. Thus, the yield can be calculated from the Eq. (2):

2 $${\text{Yield}}_{2} = \frac{y - x}{y} \times 100\,\%$$

where *x* is the final acid content of the system, and *y* is the initial acid content of the system.

#### Characterization of surfactants

##### Chemical analysis

^1^H-NMR spectra were recorded on a Bruker 500 spectrometer with D_2_O as solvent. The Fourier Transform Infrared (FTIR) spectra were collected on a Bruker Tensor-27 spectrometer using a KBr pellet, in the wavenumber range between 4000 and 400 cm^−1^.

#### Surface Tension and Critical Micelle Concentration Measurements

The surface tension of the synthetic surfactants was measured using a POWEREACH JK99C tensiometer by means of the Wilhelmy plate method at 20 °C; average values over three measurements are reported. The concentration where there is a break in the curve of surface tension versus log concentration is taken as the critical micelle concentration (CMC) [14–16].

##### Emulsifying Power

The emulsifying power of synthetic surfactant was tested according to the Chinese standard GB/T 6369-2008. First, the synthetic surfactant (0.6 g) was dissolved in distilled water (50 mL). Secondly, mineral oil (30 g) was added to the aqueous surfactant solution, and the mixture was vigorously stirred (approximately 1500 rpm) for 2 min to form an emulsion. Thirty-seconds later, phase separation appeared. After that, the emulsified mineral oil was extracted with chloroform in a separatory funnel. The optical density of the extract oil was measured on a spectrophotometer and the corresponding amount of mineral oil emulsified was calculated through the standard curve method. Emulsifying Power (EP) is calculated by Eq. (3):

3 $${\text{EP }}\left( \% \right)\, = \,\frac{m}{M} \times 100\,{\text{\% }}$$

where *m* is the weight of the mineral oil emulsified (g), and *M* is the weight of the total mineral oil added (g).

##### Lime Soap Dispersing Power

At 20 °C, aqueous sodium oleate solution (5 mL, *V*_2_, 0.1 wt %), aqueous surfactant solution (5 mL, *V*_1_, 0.25 wt %) and hard water (1 g CaCO_3_/L, 10 mL) were placed into a 50-mL cylinder. Then, deionized water (10 mL) was added to the cylinder. Next, the cylinder was plugged and was inverted 20 times successively. Finally, the cylinder was left to rest for 30 s. If there was some sediment in the mixture, more surfactant was added. When the sediment had dispersed completely, the dosage of the surfactant solution was recorded. The lime soap dispersing power (LSDP) was calculated from the Eq. (4):

4 $${\text{LSDP }}{\% } = \frac{{0.25\,\% \times V_{1} }}{{0.5\,\% \times V_{2} }} \times 100$$

where *V*_1_ and *V*_2_ represent the volume of surfactant solution (mL) and the volume of sodium oleate solution (mL), respectively.

##### Wetting Power

Contact angles were determined by a JY-82C angle instrument. The contact angle of surfactant solutions (of the same concentration) on paraffin were measured at 20 ± 10 °C [17].

## Results and Discussion

### Synthesis of Sodium Sulfoitaconate (SSI)

For convenience of purification, the sulfonate reaction was carried out with excess NaHSO_3_. The effects of molar ratios between sodium bisulfite and IA, reaction temperature and reaction time on sulfonation reaction have been investigated.

As can be seen from Table 1, larger molar ratio of IA to NaHSO_3_, higher reaction temperature and longer reaction time are favorable for the sulfonation reaction. At n(IA):n(NaHSO_3_) = 1.10:1, the sulfonation rate and the yield of SSI have the maximum values. As the molar ratio increases, the sulfonation rate decreases. A high reaction temperature is preferable for the sulfonation reaction, and the sulfonation rate is maximum at 90 °C. However, the sulfonic acid group is possibly oxidized when the temperature is above 100 °C. In the first 2 h, the sulfonation rate dramatically increases and then it slows down. Therefore, the optimum condition of SSI synthesis is found at the molar ratio of n(IA):n(NaHSO_3_) = 1.10:1, reaction temperature of 90 °C and reaction time of 5 h. In this case, the sulfonation rate is 89.19 % and the yield of sulfonation product is 83.27 %.

**Table 1:** Effect of molar ratios, reaction temperature and reaction time on sulfonation reaction

Sequence number	Molar ratios (IA:NaHSO_3_)	Reaction temperature (°C)	Reaction time (h)	Sulfonation rate (%)	Yield (%)
A1	1.05:1	90	5.0	77.36	76.42
A2	1.10:1	90	5.0	89.19	83.27
A3	1.15:1	90	5.0	71.16	71.06
A4	1.20:1	90	5.0	79.29	75.05
A5	1.25:1	90	5.0	79.38	68.94
A6	1.30:1	90	5.0	74.34	65.68
B1	1.10:1	80	5.0	71.62	69.81
B2	1.10:1	85	5.0	79.19	78.40
B3	1.10:1	90	5.0	89.19	83.27
B4	1.10:1	95	5.0	85.58	79.13
B5	1.10:1	100	5.0	79.55	55.14
B6	1.10:1	105	5.0	44.59	36.98
C1	1.10:1	90	1.0	44.31	35.77
C2	1.10:1	90	2.0	73.36	58.89
C3	1.10:1	90	3.0	84.09	79.08
C4	1.10:1	90	4.0	84.36	79.15
C5	1.10:1	90	5.0	89.19	83.27
C6	1.10:1	90	6.0	89.55	84.01

### Synthesis of Sodium *n*-Octyl Sulfoitaconate Diester (SOSID)

In the esterification reaction, the effects of molar ratios between *n*-octanol and SSI, reaction temperature, reaction time and catalyst dosage on esterification rate were investigated. An excess of *n*-octanol relative to the theoretical value is necessary to obtain the itaconate diester. As listed in Table 2 (D1 ~ D4), a larger molar ratio of *n*-octanol to SSI is favorable to synthesize diester. The esterification rate increases first and then decreases with the molar ratios of *n*-octanol to SSI increasing. The esterification rate of SSI was 94.80 % at n(*n*-octanol):n(SSI) = 2.05:1. Furthermore, the esterification rate increases as the reaction temperature increases (Table 2 (E1 ~ E4)). The esterification rate reached 94.52 and 94.80 % when the reaction temperature was 140 and 150 °C, respectively. However, the color of the resultant product darkens when the reaction temperature increases gradually, and it is hard to be decolorized. Therefore, the appropriate reaction temperature is 140 °C.

**Table 2:** Effects of synthetic conditions on esterification reaction

Sequence number	Molar ratios (*n*-octanol:SSI)	Reaction temperature (°C)	Reaction time (h)	Catalyst dosage (%)	Esterification rate (%)
D1	2.00:1	150	4.0	2.0	91.76
D2	2.05:1	150	4.0	2.0	94.80
D3	2.10:1	150	4.0	2.0	92.25
D4	2.20:1	150	4.0	2.0	89.37
E1	2.05:1	120	4.0	2.0	90.82
E2	2.05:1	130	4.0	2.0	92.46
E3	2.05:1	140	4.0	2.0	94.52
E4	2.05:1	150	4.0	2.0	94.80
F1	2.05:1	150	0.5	2.0	74.56
F2	2.05:1	150	1.5	2.0	81.80
F3	2.05:1	150	2.5	2.0	90.89
F4	2.05:1	150	3.5	2.0	95.30
F5	2.05:1	150	4.5	2.0	95.36
G1	2.05:1	140	4.0	1.0	91.98
G2	2.05:1	140	4.0	1.5	91.94
G3	2.05:1	140	4.0	2.0	94.80
G4	2.05:1	140	4.0	2.5	94.19
G5	2.05:1	140	4.0	3.0	94.58

After 3.5 h, the change of esterification rate becomes slow, which indicates the reaction time should be controlled within 4 h. In addition, the influence of *p*-toluene sulphonic acid dosage on the esterification rate can be found from Table 2 (G1 ~ G5). When the catalyst dosage was more than 2.0 wt % (Based on the weight percent of SSI), the esterification rate changes slightly.

In summary, the optimum conditions for the esterification reaction are found for a molar ratio n(*n*-octanol):n(SSI) of 2.05:1, reaction temperature of 140 °C, reaction time of 4 h and catalyst dosage of 2.0 wt %.

## Surfactant Characterization

### Structural Characterization

**Fig. 1:**
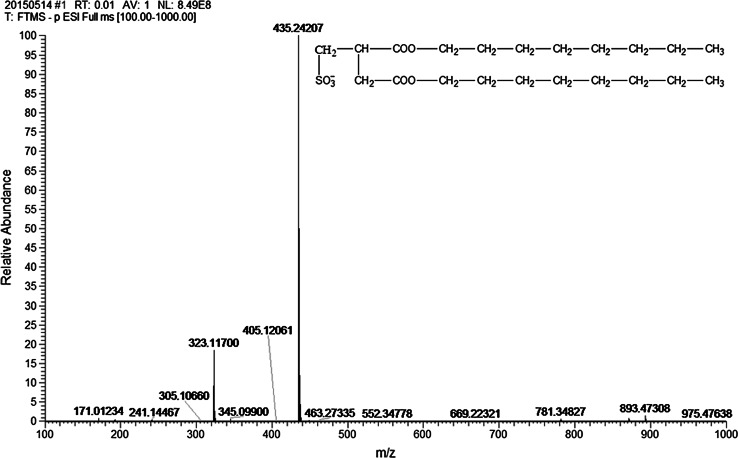
Mass-spectrogram of SOSID

The molecular structure of the prepared surfactant SOSID was determined by LC–MS analysis. As shown in Fig. 1, the assignment of 435.24207(m/e) is the representative [M-Na-(SO_3_)(COO)_2_(C_8_H_17_)_2_]^−^ fragment ion peak.

**Fig. 2:**
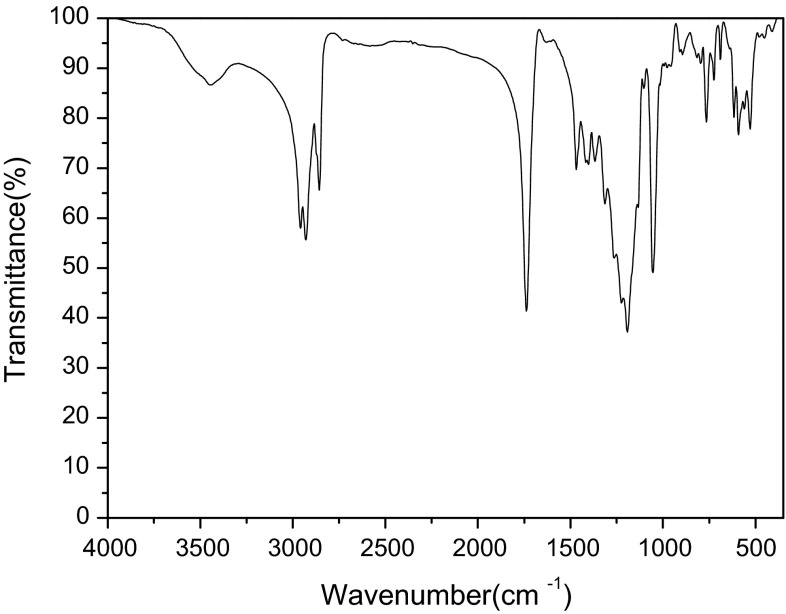
FT-IR spectrum of SOSID

**Fig. 3:**
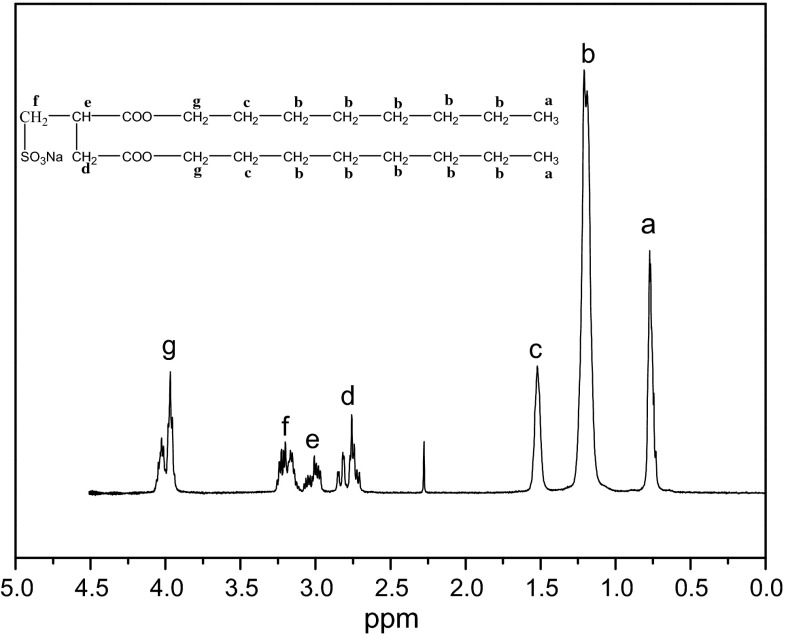
^1^H NMR spectrum of SOSID

Besides, FT-IR and ^1^H NMR analysis was conducted to confirm the structure of SOSID, as represented in Figs. 2 and 3. FT-IR spectrum of SOSID indicates that most of the characteristic intensive bands can be found at 1054 cm^−1^, 1192 cm^−1^ (SO_3_), 1737 cm^−1^ (C = O), and 3444 cm^−1^ (OH). Moreover, no stretching vibration of C = C at 1636 cm^−1^ was found, which indicated that reaction happened between IA and NaHSO_3_. From ^1^H NMR (D_2_O, 500 MHz): δ 3.95–4.05 (m, 4H); δ 3.14–3.25 (m, 2H); δ 2.97–3.06 (m, 1H); δ 2.71–2.85 (m, 2H); δ 1.52(S, 4H); δ 1.19–1.21(d, *J* = 10 Hz, 2H); δ 0.73–0.77(m, *J* = 5 Hz, 6H).

### Characterization of properties

**Fig. 4:**
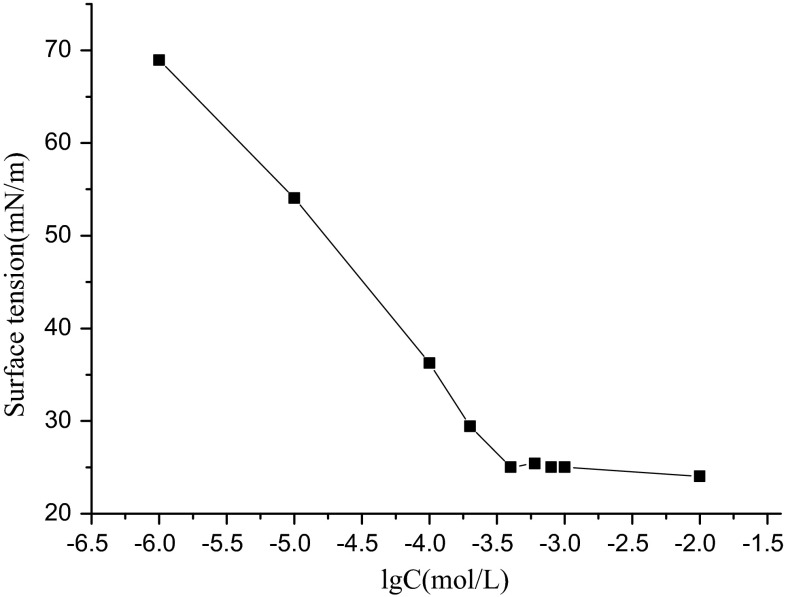
Surface tensions of aqueous SOSID solutions at different concentrations

In Fig. 4, the surface tension decreases with SOSID concentration. Obviously, there is a break point between 10^−4^ mol/L and 10^−3^ mol/L. The surface tension, *γ*, changes from 36.25–25.02 mN/m. The CMC and the surface tension at the CMC (*γ*_CMC_) are shown in Table 3. The *γ*_CMC_ is 25.02 mN/m, smaller than that of the conventional surfactants nonylphenol ethoxylate (10) ester (NP-10) and sodium dodecylbenzene sulfonate (SDBS). The CMC of the surfactant SOSID is 4.0 × 10^−4^ mol/L, smaller than that of anionic surfactant SDBS, but larger than that of NP-10.

**Table 3:** Surface-active properties of SOSID

Sample	*γ*_CMC_ (mN/m)	CMC (mol/L)	Emulsifying power (%)	LSDP (%)	Contact angle (θ)/°
SOSID^a^	25.02	4.0 × 10^−4^	30.86	>100	14.31
NP-10	37.7	(7.8–9.2) × 10^−5^	20.86	40	50.07
SDBS	39.0	1.2 × 10^−3^	46.45	>100	40.28

^a^Synthesis conditions: n(*n*-octanol):n(SSI) = 2.05:1, reaction temperature: 140 °C, reaction time: 4 h, and *p*-toluene sulphonic acid dosage 2.0 wt %

From Table 3, it is also observed that the emulsifying power of 1.0 wt % aqueous SOSID solution is 30.86 %, which is greater than that of NP-10 under the same test conditions, but inferior to the SDBS. It is easy for SOSID to be adsorbed at the surface of the mineral oil molecules and the hydrophilic head group orients towards the water phase and forms a layer of hydrophilic film because of the distinct molecular structure with two tails and one head. As an effective emulsifier, SOSID plays stabilization and protection roles in emulsion process. The LSDP value of surfactant SOSID is also shown in Table 3. In general, a surfactant including larger polar groups or more than two hydrophilic groups, such as alcohol ethoxylate sulfate and methyl ester sulfonate, presents favorable dispersing power [18]. However, only one hydrophilic group can be found in SOSID and SDBS, which reveals that they show a weaker dispersing power than NP-10.

**Fig. 5:**
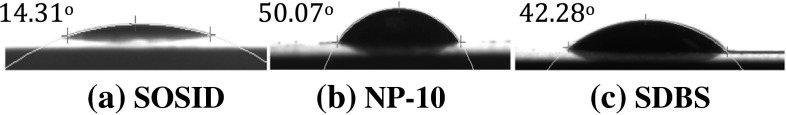
Contact angles of surfactants solutions dropped on paraffin (*C* = 1.0 × 10^−2^ mol/L)

The smaller contact angle is beneficial to wetting power. As shown in Fig. 5 and Table 3, the contact angle of SOSID is smaller than NP-10 and SDBS. Therefore, the surfactant SOSID has superior wetting capacity and may be used as a wetting agent.

## Conclusions

An anionic surfactant based on IA, NaHSO_3_ and *n*-octanol, named as SOSID, was synthesized and characterized. The sulfonation rate of the sulfonated product (SSI) is 89.4 % with the molar ratio of itaconic acid to sodium bisulfite at 1.10:1 under 90 °C for 5 h. The esterification rate of SSI is 94.52 % in the molar ratio n(*n*-octanol):n(SSI) at 2.05:1 under 140 °C for 4 h. The amount of *p*-toluenesulfonic acid catalyst is 2.0 wt % (relative to IA). The chemical structure was confirmed by FT-IR and ^1^H NMR, indicating that the objective surfactant was synthesized successfully. The physicochemical properties show that the CMC of the SOSID is 4.0 × 10^−4^ mol/L and *γ*_CMC_ is 25.02 mN/m. The SOSID had better capacity of emulsification power than NP-10 and excellent wetting capacity.
